# Intentional mind-wandering as intentional omission: the surrealist method

**DOI:** 10.1007/s11229-021-03135-2

**Published:** 2021-04-29

**Authors:** Santiago Arango-Muñoz, Juan Pablo Bermúdez

**Affiliations:** 1 Instituto de Filosofía, Universidad de Antioquia, Calle 67 N° 53-108, Of. 12-408, Medellín, Colombia; 2 Institut de philosophie, University of Neuchâtel, Espace Tilo-Frey 1, 2000 Neuchâtel, Switzerland; 3 Programa de Filosofia, Facultad de Ciencias Sociales y Humanas, Universidad Externado de Colombia, Calle 12 No 1-17 Este, Bogotá, Colombia

**Keywords:** Mind-wandering, Omission, Mental action, Creativity, Surrealism

## Abstract

Mind-wandering seems to be paradigmatically unintentional. However, experimental findings have yielded the paradoxical result that mind-wandering can also be intentional. In this paper, we first present the paradox of intentional mind-wandering and then explain intentional mind-wandering as the intentional omission to control one’s own thoughts. Finally, we present the surrealist method for artistic production to illustrate how intentional omission of control over thoughts can be deployed towards creative endeavors.

## Introduction

In the first *Manifeste du surréalisme*, André Breton (1969) presented the following definition:

Surrealism, *n*. Psychic automatism in its pure state, by which one proposes to express—verbally, by means of the written word, or in any other manner—the actual functioning of thought. Dictated by thought, in the absence of any control exercised by reason, exempt from any aesthetic or moral concern.

In the *Manifeste*, Breton recounts how he invented the surrealist method inspired by Sigmund Freud. According to the psychoanalytic method, the patient has to talk freely to the doctor and allow her mind to make all possible associations. Thus, by applying the psychoanalytic and automatic-writing methods to himself, Breton tried to produce a fast and spontaneous monologue that avoided the intrusion of critical thought, so as to generate a “*talking thought*” (Breton 1969). He let himself write frantically to express his monologue, thus producing the “*writing of thought*” (Breton 1969). The results of this endeavor were striking: he found that the texts were remarkably fertile, filled with overflowing emotion and picturesque accent, provided with striking images and burlesque sentences.

This method has interesting implications for current conceptual discussions of mind-wandering because it exemplifies the possibility of intentional mind-wandering; that is, it illustrates that people can decide to engage in and/or continue mind-wandering intentionally. According to our interpretation, surrealist artists intentionally set themselves into a position of not controlling their own stream of consciousness so that all random associations can be formed, no matter how strange or immoral they are (Breton 1969).^1^ However, the very idea of intentional mind-wandering sounds paradoxical, because it has been traditionally considered as a passive phenomenon.^2^ In a standard intentional mental action, one exerts control over one’s mental processes in order to attain a goal: e.g. when trying to solve a difficult math problem one focuses one’s attention, checks that one is following the rules of math, monitors one’s calculation, and so on. *The paradox of intentional mind-wandering* is that one seeks to intentionally eliminate or reduce control over one’s mental processes. How could the suppression of control be considered a case of intentional behavior?

In this paper, we want to offer an account that dispels the paradox of intentional mind-wandering. First, we need to talk about mind-wandering and the passivity assumption that is common in the literature (particularly in philosophy). We will do this in Sect. 2. In Sect. 3, we review some psychological studies in favor of the existence of intentional mind-wandering, and in Sect. 4 we unpack the paradox of intentional mind-wandering. In Sect. 5, we present our solution to the paradox: intentional mind-wandering can be explained, not as an intentional action, but as an intentional *omission*, specifically an omission of control over thoughts. Finally, in Sect. 6, we present the surrealist method and in Sect. 7 we analyze whether the surrealist method (understood as an instance of the intentional mind-wandering) could be better explained as an instance of daydreaming or task-shifting (a possibility suggested by Murray & Krasich, 2020), and show that the surrealist method has very different features from these mental phenomena. This suggests that the surrealist method is a clear-cut instance of intentional mind-wandering where the artist intentionally embarks on and maintains an episode of mind-wandering in order to produce an artwork.

## The passivity assumption^3^

Mind-wandering is a psychological phenomenon where the mind moves from one subject to the other in a quasi-random way. Consider, for example, the case where you get distracted from this reading by imagining your dinner. Sometimes, one realizes that one has not understood a text because one has been mind-wandering while reading. Psychologists estimate that episodes of this type comprise a large portion of our mental life (around 35–50% (Christoff et al. 2016; Killingsworth and Gilbert 2010; although see Seli et al. 2018a, b, c for a critical review)) and they have been studying their benefits (e.g. for creativity and planning) as well as their risks (for example, car accidents, deficits in understanding and reading comprehension) (Mooneyham and Schooler 2013).

The view that has become standard in the empirical mind-wandering literature (Mills et al. 2018) holds that mind-wandering happens when the subject is decoupled from tasks and external-world stimuli (Antrobus 1968; Schooler et al. 2011; Smallwood and Schooler 2006). According to this view, mind-wandering is self-generated mental activity unrelated to the current ongoing task (Konishi and Smallwood 2016; Schooler et al. 2011; Smallwood 2013; Smallwood and Schooler 2006). In a paradigm often used to test mind-wandering from this point of view, subjects are asked to perform an easy and monotonous task, such as counting breaths, and then interrupted at random intervals with a question like “describe anything in your stream of consciousness in the moments prior to the probe” (Baird et al. 2011), or “are you thinking about something other than what you are currently doing?” (Killingsworth and Gilbert 2010). The researchers then proceed to analyze the relationship between the content of the reported thought and the ongoing task. One of the main findings is that subjects tend to mind-wander more when performing easier tasks that impose less cognitive demand (Antrobus 1968; Filler and Giambra 1973).

Although the standard view does not entail that mind-wandering is non-intentional, it became common to infer from the claim that mind-wandering is task-independent to the claim that mind-wandering occurs without intention (Seli et al. 2016a, b, p. 605). Since the mind-wandering episode takes place independently from the task that the agent intends to do at the time, it is a likely interpretation of this that mind-wandering is passive, in the sense that it occurs independently of the action that the agent is intending to perform at the time: the experimental task. While several views of mind-wandering in the existing philosophical literature criticize and provide alternatives to the standard view, these alternative views tend to share with the standard view a conception of the phenomenon as characteristically passive.

The passivity assumption has been particularly influential in the philosophical literature. The discussion about how to conceptualize mind-wandering is very rich and we cannot get into the details here. We merely want to point out that the alternative accounts of mind-wandering as unguided attention or thought (Irving 2016; Irving and Thompson 2018), as absence of veto-control and meta-awareness (Metzinger 2013, 2015, 2018), and as disunified thought (Carruthers 2015; Dorsch 2015) share with the standard view the assumption that agents mind-wander when they cease to guide or control their mental processes toward goal-attainment. This is expressed differently in each account (as unguidedness, lack of meta-awareness and veto-control, or disunified thinking), and these differences have interesting theoretical consequences, but the general point remains that during mind-wandering agents are *mentally passive*, in the sense that they are not intentionally directing thought toward the attainment of a goal. Metzinger takes this passivity claim to its ultimate consequences by explicitly denying the possibility of intentional mind-wandering: “Mind-wandering can therefore be conceptualized as a form of unintentional behaviour, as an involuntary form of mental activity” (Metzinger 2015, p. 275). And so does Irving (2016, pp. 549–50):

It seems essential that mind-wandering lacks purpose; almost by definition, it contrasts with goal-directed forms of cognition like planning a trip or solving a crossword. Consider the term ‘mind-wandering’ itself. Wandering is purposeless movement…To say that someone’s mind is wandering, then, implies that her thinking is purposeless; it is not developing toward a goal or end-point.

Despite the fact that many researchers seem to agree on the fact that mind-wandering is a passive mental process, the family resemblance view developed by Seli et al. (2018a, b, c) is an exception. According to this view, mind-wandering is a heterogeneous concept that encompasses a broad range of mental phenomena with different attributes that only coincide in their family resemblance (for critical discussion see Christoff et al. 2018). This view can easily accommodate the intentionality of mind-wandering and avoid the paradox. Although this account has gained popularity in the last few years, the paradox is still relevant and worth addressing because the passivity assumption remains widespread in the philosophical literature, and also until recently in the psychological literature (for criticism in this direction see Seli et al. 2016a, b). In fact, there are some recent criticisms to the idea defended by the family resemblance view that task-unrelatedness and intentionality are compatible (Murray and Krasich 2020). This reinforces the idea that the existence of intentional mind-wandering generates a conceptual paradox.

## Intentional mind-wandering

In everyday experience intentional mind-wandering seems to be fairly common: for example, sometimes one lets one’s own mind wander because one is bored or tired of listening to a conference; sometimes one withholds one’s attention or cognitive control over one’s stream of thoughts while brainstorming to try to find a new and unexpected argument or idea, something that wouldn’t come to one’s mind in more closely controlled circumstances. Moreover, some recent empirical data show that people are aware that they mind-wander, report engaging in intentional mind-wandering (Giambra 1995; Seli et al. 2015, 2016a, b), and even seem to display a distinct psychological profile with a particular neural substrate in these cases (Golchert et al. 2017; Turnbull et al. 2019). All this suggests that we should make room in our concept of mind wandering for cases of intentional mind-wandering (Seli et al. 2018a, b, c).

The first line of evidence for intentional mind-wandering comes from some studies where people report letting their minds wander (Kane et al. 2007). Smallwood et al. (2007) asked subjects who were engaged with a task, which required to encode words and detect targets with either high or low probability, whether they (i) were focused on the task, (ii) had consciously let their minds wander, or (iii) caught themselves inadvertently mind-wandering while they were solving the task. Remarkably, they found that reports of subjects *letting their minds wander* were just as frequent as those of catching themselves mind-wandering. This suggests that subjects frequently allowed themselves to intentionally mind wander while performing a task, since they were aware of doing so before the probe and they did not exert control to stop themselves from engaging in it.

Subjects are not just aware that they mind-wander: there is a burgeoning literature providing evidence that they also report doing it intentionally (e.g., Forrin et al. 2020; Giambra, 1995; Seli et al. 2015, 2016a, b) both in the lab and in everyday life (e.g., Seli et al. 2014). Thus, Seli and his colleagues (Seli et al. 2016a, b) studied participant self-reports concerning the intentionality of their mind-wandering episodes. In their paradigm, participants had to carry out an easy or difficult variant of the sustained-attention-to-response task (SART), and were required to respond to thought probes that measured intentional and unintentional mind-wandering. They found not only that people are able to distinguish between intentional and unintentional mind-wandering, but also that there was a significant difference in the proportion of intentional mind-wandering across the easy and difficult conditions: people tended to intentionally mind-wander more frequently in easy tasks than in difficult tasks; whereas unintentional mind-wandering was more frequent in difficult than in easy tasks (Seli et al. 2016a, b).

Moreover, they found that participants can adjust their rate of mind wandering during an attention-demanding task without decreasing their performance (Seli et al. 2016b, 2018a, b, c). In this paradigm, participants were presented with an analog clock and had to press a button each time the clock pointed at 12:00. Each clock turn lasted 20 s and thought probes were presented to measure mind-wandering. The key result is that participants decreased their rate of mind-wandering when the clock was approaching 12:00. This shows that subjects can not only initiate, but strategically modulate their mind-wandering episodes, and thus supports the idea that mind-wandering can be intentional.^4^

## The paradox of intentional mind-wandering

The aforementioned results suggest not only that subjects report undergoing episodes of intentional mind-wandering, but also that there are psychologically significant differences between cases of unintentional and intentional mind-wandering. When put together, the existence of intentional mind-wandering and the passivity assumption sets the philosophical challenge of explaining how mind-wandering can be both passive in one sense and active or intentional in another.^5^*The paradox of intentional mind-wandering* is that one intentionally engages in mind-wandering only if one intentionally eliminates or reduces control over one’s mental processes, and that control is something that seems to be essential for an event to be intentional. The paradox can be expressed as follows:

(1) *The passivity assumption:* If a string of thoughts counts as an episode of mind-wandering, then the agent does not direct or control said string of thoughts toward the completion of an occurrent goal.

(2) *Intentional action:* for a given string of thoughts to constitute an intentional mental action, the agent must control *in a minimal way* said string of thoughts toward the completion of an occurrent goal.

From (1) and (2) it follows that mind-wandering cannot be an intentional mental action.^6^

Claim (1) is a formulation of the passivity assumption discussed in Sect. 2: the dynamics of mind-wandering imply that the succession of thoughts during a mind-wandering episode does not respond to a given task or external stimulus, is not guided by the agent, and is not unified by a goal.

Claim (2) merits further development. While the field of mental action is in a state of contested debate, claim (2) states a largely agreed upon condition: a string of thoughts that counts as an intentional mental action must be directed by the agent toward the attainment of a goal of hers. This applies to complex cases (e.g. stringing together sub-calculations in order to solve a mathematical problem) as well as to simple cases (e.g. remembering where you left the keys yesterday). Thus, while other conditions should be provided to fully specify which episodes of thought count as mental actions, claim (2) specifies one necessary condition.^7^ The issue, then, is that this is a condition that mind-wandering by its own nature seems unable to fulfil, given the passivity assumption. Which is why the possibility of intentional mind-wandering is paradoxical.

To further highlight the potency of this paradox, it is worth expanding on the point that mind-wandering cannot be goal-directed (claim 1). Particularly, this entails that mind-wandering episodes cannot be caused by an *intention* to mind-wander, given the functional roles that intentions play in the psychology of agency (Bratman 1987; Mele 1992).^8^ Consider particularly what Pacherie (2012) calls the “executive functions” of intentions: (1) triggering or initiating action; (2) sustaining action; and (3) guiding action.

Could an intention to mind-wander initiate, sustain and guide an episode of intentional mind-wandering? Say that an episode *M* of mind-wandering is intentional because *M* is explained by the executive role played by I(*M*), i.e. an intention relevantly linked to the production of *M*.^9^ This way of accounting for the intentionality of mind-wandering will not work. In order for an intention to trigger, sustain or guide an action, the intention must represent the action to be triggered, sustained or guided. Further, the intention’s representational content must include satisfaction conditions that specify what counts as an adequate triggering, sustainment or guidance of the action. In Pacherie’s words, “[a]n intention to A incorporates a plan for A-ing, a representation or set of representations specifying the goal of the action and how it is to be arrived at” (Pacherie 2006, p. 146). It is this set of representations of the goal and the steps that one has to take towards the goal that allows the subject to monitor and control the progress of her action. But such a set of representations cannot exist when the action in question is an episode of mind-wandering (see Murray and Krasich 2020). The content of mind-wandering episodes is quasi-random, so any representation of the expected result must be too vague to meaningfully guide action: if we cannot recognize what success amounts to, we will not be able to detect deviations from it and guide behavior accordingly.^10^ Hence we cannot represent satisfaction conditions that specify when we have successfully triggered, sustained or guided thoughts so as to constitute an instance of mind-wandering. Since we cannot represent such satisfaction conditions, it seems impossible for us to form an intention to mind-wander that can be used by agents to effectively produce (i.e. trigger, sustain or guide) an episode of mind-wandering.

Hence the *paradox of intentional mind-wandering*: intentional mind-wandering is paradoxical because the nature of intention is to represent the goals of the action, and to guide and control its execution, but in the case of mind-wandering neither the goal nor the means can be represented with sufficient specificity, and thus mind-wandering seems to be a mental process that cannot be intentionally controlled.

It could be objected that intentions to mind-wander can at least explain the intentional *initiation* of mind-wandering episodes, even if they are unable to explain how agents intentionally guide and sustain them.^11^ But this does not work either. ‘Intending to mind-wander’ is analogous to other expressions like ‘intending to fall asleep’ or ‘intending to relax’: none of these things are directly achievable by the agent; instead one has to perform other actions in order to *bring it about* that one produces the mentioned result (Mele 2009). ‘I intend to sleep’ turns out to be shorthand for ‘I intend to *bring it about that* I sleep’. Mind-wandering, like sleeping or relaxing, is something that happens to us, even though we can intentionally try to bring it about. This entails that episodes of intentional mind-wandering are not generated by intentions to mind-wander. Thus the intentionality of intentional mind-wandering must be explained by recourse to something different to intentions, decisions, and the like.

Lines of thought like these support Murray and Krasich’s (2020) argument (directed in general against the possibility of intentional mind-wandering and in particular against some of the claims of the family resemblance view) for the claim that intentional mind-wandering is impossible because task-unrelatedness and intentionality are incompatible. They claim that “an agent cannot intend to have task-unrelated thoughts, as intending to have any thought thereby makes that thought related to one’s task. Thus, the thoughts cannot be task-unrelated and so cannot constitute mind wandering in the standard sense” (Murray and Krasich 2020). We agree with Murray and Krasich that *intentions to mind-wander* cannot explain intentional mind-wandering. But this does not entail that intentional mind-wandering is impossible. What it entails is that, if intentional mind-wandering exists, its intentionality must be different from that of standard intentional actions which are originated by intentions, decisions, or similar mental states.

## Mind-wandering as intentional omission of control over thought

A possible solution to the paradox of intentional mind-wandering is to reject one of the contradictory claims (1 or 2) (as suggested by Murray and Krasich 2020). However, this strategy comes at a price: it would imply either rejecting a core component of the concept of mind-wandering (its lack of goal-directedness), or reinterpreting a series of consistent empirical findings. In this section, we propose a different solution that relies on a different understanding of the intentionality involved in (2): Mind-wandering is not an intentional action in the traditional sense of the term, but rather an *intentional omission*. Specifically, intentional mind-wandering is an intentional omission of control or guidance over one’s thoughts.

### Intentional omission

According to some researchers, mindfulness meditation can stop or, at least, reduce mind-wandering. The experienced meditator is able to focus her attention on a single target, and detect any interruption of focus, thus stopping or avoiding mind-wandering (Mrazek et al. 2012). Arguably, if one can stop or avoid mind-wandering, then one should be able to also promote that one’s mind wanders. As we will argue below, we can do that by omitting to control our own mind.

The uncontrolled nature of mind-wandering is particularly problematic for explaining how intentional mind-wandering could be possible. Some researchers may complain that given that mind-wandering is not controlled it is passive, and thus it cannot be intentional or agential. Galen Strawson highlights this issue: “the event of entertaining [content in thought] itself is not an action, any more than falling is once one has jumped off a wall” (Strawson 2003, p. 235). We agree that, in general, both entertaining a thought and falling seem not to be actions, in the same way that staying still, motionless, seems not to be an action. But in some cases, entertaining a thought, falling and staying still should be considered intentional omissions.^12^ Falling is an intentional omission, in a broad sense, if you let yourself fall while you have the possibility to stop falling. An example is when you jump from an airplane with a parachute; since you always have the possibility to release the parachute and stop falling, letting yourself fall becomes an intentional omission. In the same way, staying still is an intentional omission when it implies refraining to act (Buckareff 2018; Clarke 2010; Mele 1997; Shepherd 2014). In our view, the same is true for intentional mind-wandering: you let your mind generate random content while you have the possibility to stop it (as subjects in the psychological studies report, see Sect. 3).

But intentional mind-wandering is an intentional omission in an even stronger sense than just letting one’s mind wander while one has the chance to stop it: intentional mind-wandering is an intentional omission caused by an intention. Say Chuck is testing whether his friend Jack truly trusts him. To do this, Chuck tells Jack he will fake-punch him in his face, but Jack has to prove his trust by not moving, not even blinking when Chuck’s hand approaches. In any other occasion, if a fist rapidly approached Jack’s face, he would react by closing his eyes and moving his head. But this time, because Jack really trusts Chuck and has thus formed the intention not to blink or move, he stays still with his eyes open. This is an intentional omission *caused by an intention*, because the intention not to move was causally relevant for Jack behaving differently from the way he otherwise would have.^13^ Intentional mind-wandering is similar: one can form a certain intention, i.e. an intention not to control one’s thoughts, and this alters the pattern that one’s thoughts would tend to form had one not formed the intention. Thus, intentional mind-wandering is an intentional omission to control our thoughts in the strong sense that the omission to control thoughts is caused by the agent’s formation of an intention not to control her own thought.

In order to justify our view, we need to say more about intentional omission. For this we rely on Joshua Shepherd’s (2014) account. According to Shepherd, an omission to A is an absence of action A. If the agent forms an intention not to A, and such intention plays a role in the causal story that brought about the absence of A, then such omission is intentional. The intention not to A is causally relevant in this way when it causes the disposition not to A “by making relevant changes in the agent’s cognitive and motivational systems” (Shepherd 2014, p. 22). The intention not to A might dispose the agent to form beliefs that affect subsequent deliberation in such a way that A-ing becomes less likely; or it might alter the balance of motivation in such a way that the processes required for the occurrence of A become less likely to take place.

To clarify, not all intentions not to A make the omission of A intentional. For instance, when the agent is by default disposed towards not A-ing, an intention not to A plays no crucial causal role (thus, if Chuck has a disposition to sleep in everyday, his explicit intention not to get out of bed before 8am on Sunday plays no causal role in his not getting up early on Sunday). But intentional mind-wandering is not like that. An agent intentionally omits to control her thoughts only in cases in which her default disposition is controlling or guiding her thoughts. In that case, not controlling her thoughts is something that she does intentionally. Control omission is not a strange occurrence, since agents are naturally disposed to monitor and control their thoughts (Shea and Frith 2019), and not controlling them *at all* in many everyday behavior-guiding circumstances requires an intentional exertion of effort. As Shepherd points out: “sometimes executing an intention to omit requires a great deal of skill and effort” (2014, p. 15). This is notoriously so in the case of prolonged refrainment of control of the surrealist method (more on this below).

### Which control do we omit?

We have argued that the relevant intention in the case of intentional mind-wandering is the intention to not control one’s own thoughts. This raises a series of questions. What kind of control is the one omitted in this case? Why does its omission count as intentional? And would this intentional omission amount to a certain second-order control, i.e. control over the control of thoughts?

To move forward on these questions, briefly consider the exploration–exploitation tradeoff. In complex environments agents have to maintain a balance between *exploration* (i.e. moving towards unknown potential resources and learn about their payoffs unconstrained by task or goal representations) and *exploitation* (i.e. depleting a known resource whose payoffs are constrained by relevance to specific tasks and goals). The exploration–exploitation tradeoff has been used to characterize the difference between goal-directed thought and mind-wandering (Shepherd 2019; Sripada 2018). Goal-directed thinking involves exploitative information-search processes, whereas mind-wandering involves exploratory processes.

Researchers have proposed that there is a ‘switching mechanism’ that manages the shifts between exploration and exploitation (see Shenhav et al. 2016, 2017), thus negotiating the tradeoff between the two. The function of the switching mechanism is to optimize the use of cognitive resources by shifting from task-focused mode to exploratory mind-wandering mode and vice-versa, depending on the expected value those modes have (Kool and Botvinick 2014). Thus, after performing a given task for a while, especially if the task is cognitively undemanding, participants tend to shift attention away from the task and enter into mind-wandering or exploratory mode (e.g., Antrobus 1968; Filler and Giambra 1973). Conversely, mind-wandering episodes frequently elicit thoughts about the agent’s latent task-unrelated goals, and this elicitation makes it likely that the agent focuses on one of those goals that have now become salient (Baird et al. 2011; Klinger and Cox 1987). Mind wandering would occur when the switching mechanism disengages attention from the present task, initiating the search for an alternative task with greater value. When such a task is found, the switching mechanism would tend to go back into exploitation, focusing attention on the newly identified task and ending the mind-wandering episode (Turnbull et al. 2019).

The switching mechanism is key to illuminating the nature of intentional mind-wandering, because the causal role of the intention not to control thought is precisely to alter the functioning of this mechanism. Regularly, the switching mechanism disposes us not to stay in exploration mode for too long, since mind-wandering’s main function here is to search for secondary goals, the activation of which leads the mechanism to revert to exploitation mode. The agent’s intention not to control thought modifies the switching mechanism’s default disposition to re-engage exploitation by dampening the tendency to focus on an activated goal. That is how this intention can maintain exploration and prevent reengaging in goal-directed thinking. Additionally, forming the intention not to control thought can also initiate an episode of exploration, like when you read a text that you find boring, and thus decide to let your mind wander for a while.

This responds to our first few questions: which control do we omit by forming an intention not to control thoughts? Exploitative control, i.e. the control required to string thoughts together toward the completion of a goal. In which circumstances does this omission count as intentional? Whenever we would be by default disposed to continue in or revert to exploitation, but our having formed an intention not to control thoughts modifies that default disposition. In these cases, said intention is causally relevant for either initiating or maintaining an episode of exploratory thought, and therefore such initiation and maintenance are intentional.

One question remains: does this intentional omission amount to a kind of second-order control, i.e. does it amount to controlling the control of thoughts? This can indeed be so, particularly in the intentional maintenance of mind-wandering. For such maintenance requires monitoring whether attention is focusing on a goal, in order to correct it. When you visit a new town and want to explore it, sometimes you walk through its streets willingly trying to get lost, simply wandering with no destination, letting the city surprise you. If you suddenly find yourself walking through the same streets repeatedly, you will notice that you are not succeeding in freely exploring the town, and might try to break the cycle by e.g. taking a different turn at an intersection. Similarly, if a thinker wants to omit control over her thoughts, simply trying to get lost and freely wander through patterns of association, if she notices herself repeatedly visiting the same thoughts she will recognize that she is failing in her intention to get lost in thought, and can then try to correct this by exploring different branches of a tree of associations. Insofar as intentionally maintaining exploration requires this kind of monitoring and correction, we must conclude that it does require a kind of second-order control. Such monitoring capacity could be characterized as a form of metacognition, since it would allow us to monitor and regulate the cognitive system’s mental dynamics (exploration/exploitation).^14^

To sum up, an *intentional omission* of action A is a case where, by forming an intention not to A, the agent generates dispositions that play a causal role in the absence of A occurring. Accordingly, *intentional mind-wandering* is a phenomenon in which, by forming the intention not to control thoughts, the agent generates dispositions that play a causal role in the absence of control over an episode of her thoughts. These dispositions are modifications of the switching mechanism’s tendency to return from exploration to exploitation, and are possible thanks to the capacity to monitor and distinguish between goal-constrained (i.e. controlled) and goal-unconstrained (i.e. mind-wandering) dynamics of thought. This is how intentional mind-wandering can be explained as a case of intentional omission.

## The surrealist method as an instance of intentional mind-wandering

The type of intentional omission of control presented in the previous section is the kind of process that surrealist artists turned into a method and refined to the level of a skill. André Breton invented the surrealist method inspired by the automatic writing technique developed by psychiatrists (e.g., Myers, Flournoy, Richet and Janet [see Koustaal 1992 for a review of the early studies on automatic writing]) and Freud’s psychoanalytic method (Maclagan 2014; Polizzotti 2009). During his medical studies, Breton learned about the work of the early psychiatrists and became interested in their studies on automatic writing experiments, in which subjects were instructed to place their hand on a planchette (a rolling hand-holder with a pen attached) and allow it to write freely while they were doing another task that required all their attention such as reading a novel, reciting a poem, or conversing with another person (see Koustaal 1992 for a review of these studies). According to the psychoanalytic method, the patient had to freely talk to the therapist and let her mind make all possible associations. By applying the combination of the psychoanalytic method and the automatic writing technique to himself, Breton sought to produce a fast and spontaneous monologue that avoided the intrusion of critical thought, so as to generate “*spoken thought*” (Breton, 1969). He let himself write frantically to express his monologue, thus producing the “*writing of thought*”^15^ (Breton 1969), something that would work only if the deliberate mind was “willfully and concentratedly passive” (Stockwell 2017).^16^

Thus, according to the surrealist method, the artist should actively prevent herself from interrupting or directing her spontaneous stream of consciousness so that random associations could be formed and pursued without inhibition (Breton 1969; Stockwell 2017). This was clearly stated by surrealist poet David Gascoyne:

The discipline involved in automatic writing is that of vigilantly resisting the temptation to interrupt the stream of consciousness, or to interfere with or in any way alter *post facto* the results obtained ‘with laudable disdain as regards their literary quality’ [in Breton’s phrase] (Gascoyne, in Breton, Eluard and Soupault 1997, p. 48).

This ability to vigilantly allow the uninterrupted flow of consciousness corresponds to the omission of control enabled by the agent’s metacognitive capacity to monitor the dynamics of thought, so as to maintain exploratory dynamics and avoid falling into exploitative dynamics. It also underscores that what matters in intentional mind-wandering is not only the initiation, but also the maintenance and the guidance of the mind-wandering episode.

While almost anyone can try to avoid controlling her thoughts and engage in automatic writing, it is not as easy as it would initially seem. As Dorsch pointed out, “it is rather difficult for us to remain purely passive for very long with respect to our mental lives” (2015, p. 804). In fact, people are so resistant to mind-wandering that many are even willing to give themselves electric shocks rather than spend fifteen minutes alone with their thoughts (Wilson et al. 2014). The switching mechanism described above accounts for this difficulty in sustaining mind-wandering episodes. The surrealist artists realized that some people are more skilled than others at entering and remaining in the trance-like state conducive to automatic writing. Robert Desnos and René Crevel were considered particularly skillful automatic writers; they were very good at self-inducing and sustaining an automatic writing ‘trance’ (Cf. Stockwell 2017, p. 55).

The surrealist method exemplifies how mind-wandering can be purposive even if its goals are underdetermined or vague. The surrealist artist attempts to mind-wander in order to create an artistic product, but she does so with no prior conception of what she will create. She may know that she aims to write a text, but she may not know if it will concern a skull, the paradise, or just words.^17^ This lack of a goal representation exemplifies why intentional mind-wandering cannot occur under the standard form of agential control based on an intention to A (discussed in Sect. 4), where the intention represents A’s satisfaction conditions over the production of the artwork; because she does not know what exactly she is creating—in fact, it is a necessary aspect of the surrealist method that the product’s characteristics remain radically under-determined.

## The surrealist method and its narrative structure: Between mind-wandering and daydreaming

Given that the surrealist method seems to be intentional and that mind-wandering has traditionally been considered by many as non-intentional, a skeptic with regard to intentional mind-wandering may argue that that the surrealist method is not really an instance of mind-wandering but rather an instance of “focused daydreaming” (episodes of imaginative thought constrained by a purpose, e.g. imagining one’s future vacation to the Caribbean coast) or “task-shifting” (where attention focuses away from externally-defined task and towards an internally-defined one, like planning for an essay you have to write or deciding what to cook for dinner tonight) (Murray and Krasich 2020). Crucially, daydreaming and internal goal-directed thought are not cases of mind-wandering: they are both forms of goal-directed thinking.

This skeptical challenge is consistent with the above description of the switching mechanism: when the external task’s expected value is too low, the mechanism can spontaneously trigger intentional mind-wandering to search for an alternative goal, and can focus on it once it has been found. These episodes of distraction could be experienced as deliberate, since both daydreaming and motivated task-switching gravitate towards the agent’s secondary goals.

We suspect that there is no experimental evidence to solve this issue yet. So we take a different route to argue that the surrealist method is an instance of intentional mind-wandering and not a case of focused daydreaming or task shifting.

Dorsch (2015) characterizes “focused daydreaming” episodes as open-ended imaginative projects that possess a purpose; in focused daydreaming the subject voluntarily forms an imaginary representation with specific contents and a clear narrative structure guided by the purpose of her project. His example of daydreaming is the representation of a climb of Mount Everest: there are many forms of representing this fact, but the purpose determines that you do it and how you do it (i.e. it fixes satisfaction conditions). In contrast, Dorsch presents mind-wandering as a “sequence of simple, associatively linked mental episodes” that appear in the mind in a passive and uncontrolled way. Thus, Dorsch distinguishes “focused daydreaming” from mind-wandering in that only the former has a representational purpose (that fixes satisfaction conditions), a clear narrative structure (more about this below), intelligibility, and unity.^18^

The intentional mind-wandering at play in the surrealist method might seem similar to daydreaming because it is a purposeful mental process (its purpose is to produce an uninterrupted stream of thought) and it implies covert agency (withholding control over one’s stream of thoughts), but it is crucially different: intentional mind-wandering lacks a representational purpose that guides and constrains imagery production (as we said, in cases like the surrealist method, the goal it responds to must be undetermined), establishing a clear narrative structure (narration and order are secondary in surrealist artworks). In fact, the presence of such constraints and of a narrative structure would be perceived as an error, and the surrealist artist would guide her thought away from them in order to maintain a wider, unconstrained search strategy. The concept of focused daydreaming cannot capture this set of features. That is why we need to include and accept intentional mind-wandering as a distinct concept that aims to capture a different phenomenon, of the kind illustrated by the surrealist method.

Here it is important to compare the narrative structure of the surrealist method’s products with those of focused daydreaming. According to Dorsch, focused daydreaming’s narrative structure has the following features: regularity (imagined events behave in a regular way), development (there is some unfolding of events: “movement, metamorphosis, maturation, or revolution”), persistence of particular entities (i.e., referential stability), perspective (events are presented from the narrator’s single perspective), temporal order (one thing is represented as happening either simultaneous to or after another). In contrast, the products of the surrealist method lack any of these features or possess them in a very weak sense (see Stockwell 2017). Surrealist texts systematically break any regularity: almost nothing in the surrealist world behaves as it does in daily life. Consider the following example:

Towards four o’clock that day a very tall man was crossing the bridge which links up the various islands. The bells or the trees were ringing. He thought he could hear the voices of his friends: ‘The bureau of lazy excursions is on the right,’ someone called to him, ‘and on Saturday the painter will be writing to you.’ The neighbours of the solitudes leant out and the wheezing of the street-lamps could be heard all night long. The erratic house loses its blood. We all love conflagrations; when the sky changes colour, it is a dead man’s passing. What better could one hope for? Another man in front of a perfumer’s shop was listening to the rollings of a distant drum-beat. The night that was hovering above his head came down to perch on his shoulders. Conventional fans were up for sale: they weren’t producing fruit any more. Without knowing the results people were running in the direction of the maritime inlets. The desperate clocks were telling the beads of a rosary. The virtuous hives were organising themselves. There was no one passing near those main avenues that are the strength of towns. A single storm was sufficient. Far away or close up the damp beauty of prisons went unrecognized. The best shelters are railway stations since travellers never know which route to pursue. It could be read in the lines of palms that the most fragrant pledges of fidelity have no future. What can we do with the children with well-developed muscles? The warm blood of bees is preserved in mineral water bottles. Sincerities have never been seen unmasked. Well-known men lose their lives in the recklessness of those fine houses which set hearts a-flutter.

(Breton et al. 1997, p. 115).

This passage shows that despite the fact that surrealist texts are syntactically ordinary, they present semantic deflection and dislocation in attributing to objects properties that they normally lack, such as attributing faith to clocks (Stockwell 2017, pp. 59–63). Surrealist texts (especially those which have narrative appearance) have weak referential stability: they are characterized by the use of indirect propositions and indirect co-reference (by means of lexical and anaphoric repetition; see Stockwell (2017) for an analysis). Because of this feature, surrealist texts are also characterized by lacking a clear development and temporal order: the reader does not always know or understand what is going on: images just pass by without any clear connection or logical sequence.

Insofar as surrealist works like these characterize intentional mind-wandering, the aforementioned properties of these texts (lack of temporal order, narrative structure and referential stability) also clarify why intentional mind-wandering is not identifiable with motivated task-switching, as Murray and Krasich (2020) have suggested. This suggestion corresponds with the current concerns hypothesis, according to which the mind wanders when there is a greater incentive to focus on internal goals than on external tasks (Klinger 1987; Klinger et al. 1980). We share the worry that many of the self-reports obtained in experimental studies might be explained as belonging to motivated task-switching (or focused daydreaming) rather than to intentional mind-wandering. Further studies will be needed to solve this ambiguity. However, we believe that the surrealist variety of intentional mind-wandering does not suffer from such ambiguity, because it lacks the minimal narrative and referential structures that would be necessary for the agent to pursue any goal or task, including those internally generated.

In fact, given its deliberate search for contrast and incoherence, the surrealist method seems to exemplify intentionally taking the mind’s exploration mode to its extreme: casting its net as widely as possible to capture all available routes of thought, purposefully allowing the mind to meander aimlessly through associations, and maintaining this state for as long as possible before returning to narrower, more structured forms of thinking. The surrealist method, therefore, constitutes the distillation of intentional mind-wandering.

## Conclusion

The main result of our analysis is that subjects can intentionally mind-wander by omitting to control their thoughts. That is, by forming the intention not to control thought, the agent generates dispositions that play a causal role in the production of an episode of unguided thinking. This disposition can be understood as having an influence on the switching mechanism that regulates the transitions between exploration and exploitation dynamics, so that the mind can stick to the exploratory dynamics instead of reverting to more exploitative, goal-directed thought dynamics.

Surrealist artists realized that they were able to intentionally mind-wander and made of this a full-fledged creative method. They thereby harnessed the generative power of mind wandering towards the fulfilment of their creative goals. Crucially, contrary to other forms of mental intentionality, this was possible not by specifying a representation of the goal, but rather by purposefully and vigilantly avoiding to represent *any* goal.

Our account of intentional mind-wandering leaves some open questions for future research: What are the precise psychological and neural mechanisms that enable the switch between exploration and exploitation? Exciting new research associates this switching mechanism with the functioning of the dorsolateral prefrontal cortex (Turnbull et al. 2019), and research in this direction promises to shed light on the switching mechanism’s specific value functions. Another empirical question to be resolved is whether, and how often, the radical exploratory dynamics that we have identified at work in surrealist intentional mind-wandering occurs in everyday life, and whether participants in the lab can be led to produce such dynamics by instructing them to omit control over their thoughts. Interesting philosophical questions also emerge surrounding intentional *mental* omissions: How are intentional mental omissions related to bodily omissions? What other kinds of intentional mental omissions are there, and how can we provide a general account of them?

## Footnotes

1 Arguably, this may happen in other kinds of creative production as well. We have chosen surrealism because surrealists were self-aware of the psychology of their creative process (i.e., they had a clear conception of the methods they wanted to use whenever they created artworks, and of the psychological mechanisms they were appealing to) in a way that is not so evidently present in other artists or artistic movements.

2 It is worth pointing out that we do not claim that all or most cases of mind-wandering are intentional, nor that all instances of mind-wandering are as chaotic as the artwork created by some surrealists. Our view leaves ample room for cases of unintentional mind-wandering and degrees of control and freedom in mind-wandering, so that the concept can encompass different forms of the phenomenon.

3 The assumption that mind-wandering is necessarily passive is not universally accepted among psychologists. In particular, those working on the ‘family resemblance view’ of mind-wandering accept that it can be active. However, even if one accepts that mind-wandering can be active and intentional, the task remains of explaining how agents can intentionally generate the absence of goal-directed control over thoughts. In other words, even if you accept the possibility of active mind-wandering, there still remains the question what psychological processes make it possible for agents to intentionally mind-wander. Thus, if the reader accepts that mind-wandering can be active and intentional, s/he can feel free to jump straight to Sect. 5 (where we offer our omission-based account of intentional mind-wandering) and 6 (where we present the surrealist method to illustrate our account). If the reader grants some plausibility to the idea that mind-wandering is a passive phenomenon, we encourage him/her to read on.

4 A reviewer pointed out that the reported data only show that subjects can *decide* to mind-wander, but not that they can *maintain* it. We agree with this and consider that more experimental work is needed to address the question of maintenance. With that in mind, however, we consider that the surrealist method provides initial support for this idea. We address this issue further in Sect. 6.

5 This paradox resembles Irving’s “Puzzle of the purposeful wanderer” (2016). However, Irving’s puzzle has more to do with the fact that one’s episodes of mind-wandering are sometimes caused and motivated by our secondary goals, rather than to the fact that one intentionally starts or maintains an episode of mind-wandering. The paradox is also very closely related with Murray and Krasich’s *Puzzle of Wilful Wandering [PWW]*, which shows that the standard view is incompatible with the existence of intentional mind-wandering, and leads to the conclusion that “an agent cannot intend to have task-unrelated thoughts, as intending to have any thought thereby makes that thought related to one’s task. Thus, the thoughts cannot be task-unrelated and so cannot constitute mind wandering in the standard sense” (Murray and Krasich 2020). However, our paradox has a broader scope than *PWW*, since it applies not only to the standard view, but also to any view of mind-wandering that shares the passivity assumption.

6 We are trying to capture here a minimal criterion for calling something an intentional action. Thus, we do not consider “having a prior intention” or “proximal intention” to A-ing as necessary for being in minimal control of A-ing. Rather, having minimal control over one’s A-ing requires being able to guide or direct the processes constitutive of A. Correcting one’s posture if one is losing balance, or stopping oneself from saying something nasty to someone are cases of control in the sense we have in mind here.

7 Strawson (2003) has famously argued that the realm of mental action is limited to the prefatory stages of setting the stage for mental content automatically to appear before consciousness. According to him, the production of the relevant content is solely dependent on subpersonal processes and not agentive; and the preparation of the mind for the right content to appear is the sole locus of mental agency. Now, instances of said preparation also count as cases of what we call ‘controlling a string of thoughts’, in the sense that the preparation is a part of a mental process that includes the intention to bring about content C, the preparatory stages appropriate for C to arise (e.g. clearing attention of distracting elements, focusing on C-relevant thoughts), the emergence of C, and the evaluation whether C satisfies the intention’s success conditions. Thus, even minimal views of mental action accept our claim (2).

8 An anonymous reviewer raised the possibility that the intentionality of intentional mind-wandering might be explained by a decision to mind-wander. If ‘decision’ is understood as the process of selecting one option among several processes which culminates in the formation of an intention (e.g., Mele 2017), then the following arguments about the intention to mind-wander also apply to the decision to mind-wander.

9 I(*M*) could be the intention directly to produce *M*, or the intention to perform another action that relevantly requires the production of *M* as its means or as an expected side-effect (cf. Murray and Krasich 2020). Some researchers claim that agents can produce intentional actions without the need to form an intention (Bumpus 2001; Herdova 2018). Here we remain outside of the debate and focus on cases of intentional actions produced by intentions; whether other cases exist merits separate treatment.

10 Someone may object that, in the case of intentional mind-wandering, the subject can rely on vague projects: “projects whose completion does not occur at any particular or definite point or moment” (Tenenbaum and Raffman 2012). Projects like writing a book are vague in this sense, since the order of steps and their moment of realization are not defined by the project itself. However, intentions to mind-wander do not fall within the ‘vague projects’ category. Vague projects are vague about the timing and ordering of the means to achieve the goal, but are clear about what those means are (e.g. it is clear that writing a book requires reading relevant literature, typing words, and revising drafts). By contrast, the intention to mind-wander is vague about the very nature of the means whereby one could satisfy it. Hence one can guide action using an intention to complete a vague plan, but not using an intention to mind-wander.

11 We thank an anonymous reviewer for suggesting this objection.

12 Another example is fasting. Not eating is not normally an intentional action, but fasting is clearly an intentional omission.

13 Despite the fact that Chuck’s case is the inhibition of a reflex, intentional omission happens whenever the agent omits doing A when she has a strong disposition to do A, and the intention not to A is causally relevant to her not performing A. (The strong disposition may be due to a whole range of situations, like having acquired a habit, having formed a belief, a judgment, a desire, or a thought, not just a reflex).

14 For an account of intentional mind-wandering along these lines, as relying on a capacity for ‘meta-control’, see Irving (Manuscript). For a discussion of metacontrol as the capacity to negotiate the exploration–exploitation tradeoff, see Musslick and Cohen (2020).

15 "Completely occupied as I still was with Freud at that time, and familiar as I was with his methods of examination which I had had some slight occasion to use on some patients during the war, I resolved to obtain from myself what we were trying to obtain from them, namely, a monologue spoken as rapidly as possible without any intervention on the part of the critical faculties, a monologue consequently unencumbered by the slightest inhibition and which was, as closely as possible, akin to spoken thought" (Breton 1969: 22–3, original emphasis).

16 It is important to notice that the surrealist method evolved over time. Here we are mainly interested in the original version developed by Breton and the first surrealists around 1920, in which psychic spontaneous automatism was the key element (seen at work, e.g., in Breton’s *The magnetic fields, Mourning for mourning,* and his poems of this period). Although the results of the surrealist method seemed initially to be very fertile and aesthetically promising, surrealists soon discovered the shortcomings of the method (it led to overly chaotic works that lacked any sort of narrative or thematic unity) and abandoned it. Thus, other surrealists set the automatism part aside or reduce it in order to attain less chaotic poems or artworks (Stockwell 2017). This is particularly clear if one compares Breton’s verses with some of Desnos (see Stockwell 2017, p. 64 for an analysis), or André Masson’s automatic drawings with Dali’s paintings. This reveals an interesting limit to the connection between intentional mind wandering and creativity. Creative thinking is said to skillfully engage both the spontaneous “rough sketching” of ideas and the deliberate and evaluative critique of said sketches (Fox and Christoff 2014). Intentional mind wandering in its pure form, as deployed by the original surrealist method, enables the rough sketching, but cannot account for the skillful critiquing usually required to produce compelling creative works. The broader links between creativity and intentional mind wandering deserve independent exploration.

17 Consider the following verses of Robert Desnos: *O mon crâne, étoile de nacre qui s’étoile* [Oh my skull, mother of pearl which fades out]. *Au paradis des diamants les carats sont des amants …* [In the paradise of diamonds carats are lovers …]. *Mots, êtes-vous des mythes et pareils aux myrtes des morts?* [Words, are you myths and similar to the myrtles of the dead?] (cited in Stockwell 2017, p. 55).

18 Although there are also reported cases of unintentional daydreaming, these cases, unlike mind-wandering, also have clear narrative structure, intelligibility and unity (Klinger et al. 2018, pp. 223–224).
